# *TCF12* microdeletion in a 72‐year‐old woman with intellectual disability

**DOI:** 10.1002/ajmg.a.37083

**Published:** 2015-04-13

**Authors:** Juliette Piard, Virginie Rozé, Alain Czorny, Marion Lenoir, Mylène Valduga, Aimée L. Fenwick, Andrew O. M. Wilkie, Lionel Van Maldergem

**Affiliations:** ^1^ Centre de génétique humaineUniversité de Franche‐ComtéBesançonFrance; ^2^ Laboratoire de génétiquehistologie et biologie de la reproductionUniversité de Franche‐ComtéBesançonFrance; ^3^ Service de neurochirurgieUniversité de Franche‐ComtéBesançonFrance; ^4^ Service de radiologie pédiatriqueUniversité de Franche‐ComtéBesançonFrance; ^5^ Laboratoire de génétiqueUniversité de NancyNancyFrance; ^6^ Weatherall Institute of Molecular MedicineUniversity of OxfordJohn Radcliffe HospitalHeadingtonOxfordUK

**Keywords:** *TCF12*, microdeletion, intellectual disability, craniosynostosis, dysmorphism

## Abstract

Heterozygous mutations in *TCF12* were recently identified as an important cause of craniosynostosis. In the original series, 14% of patients with a mutation in *TCF12* had significant developmental delay or learning disability. We report on the first case of *TCF12* microdeletion, detected by array‐comparative genomic hybridization, in a 72‐year‐old patient presenting with intellectual deficiency and dysmorphism. Multiplex ligation‐dependent probe amplification analysis indicated that exon 19, encoding the functionally important basic helix‐loop‐helix domain, was included in the deleted segment in addition to exon 20. We postulate that the *TCF12* microdeletion is responsible for this patient's intellectual deficiency and facial phenotype. © 2015 The Authors. *American Journal of Medical Genetics Part A* Published by Wiley Periodicals, Inc.

## INTRODUCTION

Heterozygous mutations in *TCF12* were recently identified as a major cause of craniosynostosis [Sharma et al., 2013], mainly in patients with bilateral (32%), and unilateral (10%) coronal synostosis who did not previously have a molecular genetic diagnosis. All but one of the 38 mutations identified were located in *TCF12* exons 10–19. Exon 20 does not contain any recognized protein motif and exon 21 is non‐coding. Mutations were predominantly of the nonsense, frameshift, or splicing type and rarely missense; a spectrum suggestive of a loss‐of‐function (haploinsufficiency) mechanism. In the original report, no whole exon deletion of *TCF12* was identified using MLPA analysis (n = 226), suggesting that *TCF12* microdeletion is not a frequent cause of isolated craniosynostosis. A significant number of mutations were observed in close relatives who did not have either craniosynostosis or intellectual disability, indicating marked non‐penetrance (53%). We report here on a 72‐year‐old patient presenting with intellectual disability, dysmorphism, and a *TCF12* microdeletion detected by array‐comparative genomic hybridization.

## CLINICAL REPORT

The proband is a 72‐year‐old female evaluated at the request of her brother because of intellectual disability (ID), in order to provide genetic counselling to the family. She was born to unrelated parents. Her mother had a stillborn child. Some relatives were relatively short; one of her brothers was 158 cm tall (−2.3 SD) and her father was reported to be 152 cm tall (−3.8 SD). One of her sisters and a nephew had a history of seizures.

In the past medical history, the proband presented in infancy with delayed milestones. She walked without assistance at 4 years. She pronounced her first words at 7 years and required special education. She possesses a basic knowledge of reading and is able to write elementary sentences. She remained dependent on family members for organization of her daily life. In early adulthood, she was recognized as a disabled person, being placed under the supervision of a guardian. Despite the fact she was a slow learner and had limited autonomy, she worked as a cleaning lady in an institution, and had a child. Her healthy son had nine children of normal intelligence. A diagnosis of Turner syndrome was made in one of them. At the end of the fifth decade, she was diagnosed with moderate hearing loss, similar to two of her brothers, and required hearing aids. She had an episode of acute pancreatitis of unknown etiology at the age of 69 years.

**Figure 1:**
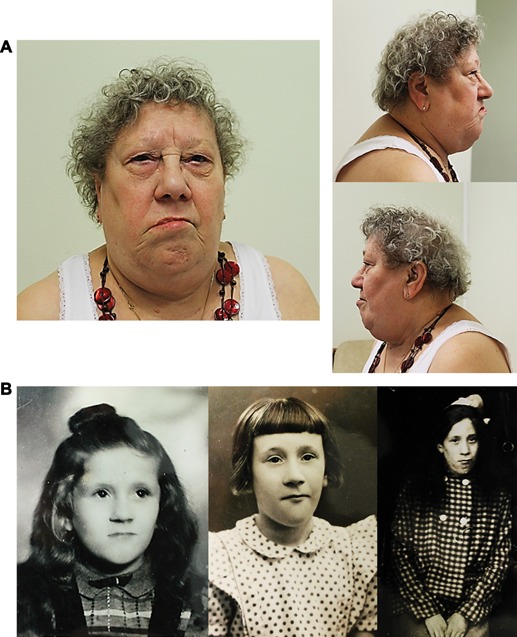
(A and B): Photographs of the patient at 72 years (A) and during childhood (B) showing facial dysmorphism. Note small eyes, thin upper lip, prominent chin and facial asymmetry with left facial nerve palsy.

When examined at the age of 72 years, short stature was noted (146 cm; −3.5 SD), with relative obesity (56.5 kg; +0.5 SD), and an occipitofrontal circumference at the lower limit of normal (52.5 cm; −2 SD). She had distinctive facial features including apparently small eyes, a thin upper lip, a prominent chin, and facial asymmetry with left central facial nerve palsy (Fig. 1A). There was a high palate. Her facial dysmorphism was better appreciated on photographs taken during childhood (Fig. 1B).

The karyotype was 46,XX (lymphocytes, G‐banding). A search for the fragile‐X trinucleotide expansion was negative. Brain magnetic resonance imaging (MRI) was normal. 3D skull computed tomography indicated thickening of the cranial vault.

**Figure 2:**
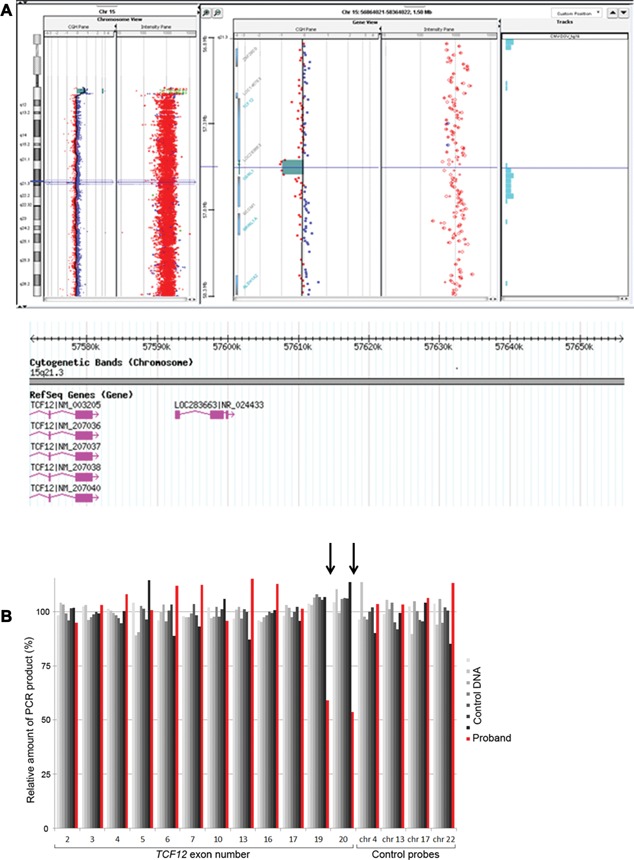
(A and B): Identification of *TCF12* microdeletion. A: Result of aCGH analysis of chromosome 15 with Human genome CGH Microarray 180K (Agilent) showing a deletion at 15q21.3 with 5 consecutive probes showing reduced copy number, encompassing exons 20‐21 of the TCF12 gene and LINC00926 gene. RefSeq genes included in the deleted interval from DGV (Database of Genomic Variants) are shown (http://dgv.tcag.ca/) in pink color. B: *TCF12* MLPA analysis. Comparison is made between signals obtained from the proband's DNA (red) and seven normal control samples (shaded grey). Twelve different exons of *TCF12* and four control loci were analyzed in this experiment. In the proband's sample the signals for exons 19 and 20 are 59% and 54% of normal values respectively, indicating a heterozygous deletion of these exons. Exon 17 and more 5′ exons of *TCF12* exhibit a normal dosage (95‐115%).

Array‐CGH analysis (aCGH), performed with an Agilent Human Genome CGH Microarray Kit 180 k (Agilent Technologies Inc., Santa Clara, CA) with a resolution of ∼25 Kb, revealed a small (84–121 kb) microdeletion of 15q21.3 encompassing the terminal coding exon, and 3′ untranslated region (UTR) of the *TCF12* gene (exons 20 and 21) and *LINC00926*, designated ISCN 2013: arr[hg19] 15q21.3(57,571,980‐57,656,064) x1 (Fig. 2A). Array‐CGH was performed according to the manufacturer's instructions. The data were analyzed by Agilent Cytogenomics software with the statistical algorithm ADM‐2, using 3‐probe minimum aberration call. The Database of Genomic Variants (http://projects.tcag.ca/variation/) was used to compare findings to previously reported studies. Coordinates of copy number variations are based on the GRCh37/hg19 assembly.

Quantitative PCR (qPCR) analysis confirmed the 15q21.3 microdeletion. Specific target sequences were selected using Primer 3 software (http://primer3plus.com). Two primer pairs were created (first pair; forward: 5′ GGTATGCTGGCAGATTGCTT 3′, reverse: 5′ TGTTAGGCACCCAATGTGAA 3′ located between nucleotides 57,581,340 and 57,581,405 [hg19] and second pair forward: 5′ CACAGTTTTGCTCAGGGTCA 3′, reverse 5′ AGCTCTGGTGAGACCAGCAT 3′ located between nucleotides 57,646,304 and 57,646,370 [hg19]). A control amplicon was used with the same parameters in *RPPH1* (localization: 14q11.2; size *ca* 60pb; Tm 60°C). qPCR assays were performed using SYBR Green (Quiagen, Holden, Germany) and analysed on a LightCycler^®^ 480 Real‐Time PCR System (Roche, Basel, Switzerland).

Multiplex ligation‐dependent probe amplification (MLPA) analysis was performed as described [Sharma et al., 2013] and demonstrated that, of the exons analyzed, the deleted portion of *TCF12* was restricted to exons 19–20 (Fig. 2B).

Her parents being deceased at the time of evaluation, we were unable to demonstrate its de novo occurrence. A qPCR search for a similar microdeletion in two of her brothers, her sister and her son was negative (data not shown).

## DISCUSSION

Heterozygous mutations in *TCF12* were recently identified as a major cause of craniosynostosis [Sharma et al., 2013]. Two recent reports [Di Rocco et al., 2014; Paumard‐Hernandez et al., 2014] have further defined the phenotypic spectrum of *TCF12* mutations. These reports confirm incomplete penetrance and show that coronal synostosis can be associated with additional features including facial asymmetry, ear anomalies (prominent ear crus, low set ears), ptosis of the eyelids, hearing loss, and hand/foot anomalies (brachydactyly and syndactyly). Mutations identified by Di Rocco et al. were frameshift (3) or missense (1), all included in sequences encoding either the activation domain 2, or the basic helix‐loop‐helix (bHLH) domains [Di Rocco et al., 2014]. Mutations reported by Paumard‐Hernandez et al. were nonsense (2), splicing (1), frameshift (1), or missense (1) and three of them were included in the activation domain 2 [Paumard‐Hernandez et al., 2014].

In our patient, array‐CGH performed in the context of a workup for intellectual deficiency identified a small 15q21.3 microdeletion encompassing a single gene referenced in the OMIM database: *TCF12*. In order to define precisely the deleted interval, MLPA analysis was performed. This indicated that the critical exon 19, encoding the functionally important bHLH domain, was included in the deleted segment in addition to exon 20 (Fig. 2B). Therefore bHLH domain would be deleted and *TCF12* partial microdeletion is likely to be pathogenic in the present case. The other gene included in the deleted interval is a long intergenic non‐protein coding RNA (*LINC00926*), whose function is still unknown. Its contribution to the phenotype is currently uncertain.

At clinical examination our patient had a normal skull shape and there was no evidence of untreated craniosynostosis on examination or on 3D cranial CT. However we do not have adequate documentation from her childhood to exclude the possibility of premature fusion of her cranial sutures.

We postulate that *TCF12* microdeletion is responsible for the intellectual deficiency observed in our patient. There have been occasional reports of larger chromosomal deletions encompassing *TCF12* in patients with craniosynostosis and intellectual disability [Fukushima et al., 1990; Shur et al., 2003; Hiraki et al., 2008; Le Tanno et al., 2014]. However, these patients had larger deletions with a high number of potential ID genes included in the deleted interval, making evaluation of a role for *TCF12* in the phenotype difficult. The 3.6 Mb heterozygous deletion reported by Le Tanno et al. contains more than 20 known protein‐coding genes including *TCF12*. The coronal craniosynostosis described in the patient can very likely be attributed to *TCF12* haploinsufficiency. Involvement of another gene included in the deleted interval to account for ID of his patient remains speculative [Le Tanno et al., 2014]. Interestingly, a few additional patients with large 15q21q22 deletion presenting with ID without craniosynostosis have been also reported [Tempesta et al., 2008; Yamamoto et al., 2014].

Fourteen percent (10/72) of patients with *TCF12* mutation reported by Sharma et al. [2013] had significant developmental delay or learning disability, and two an autism spectrum disorder (ASD). Two out of nine (22%) patients reported by Paumard‐Hernandez et al. [2014] presented with ID and ASD. This is suggestive of a role for *TCF12* in neurodevelopment. The conclusions of these two series, alongside with our own report, are in contrast to Di Rocco et al. [2014] who reported that all of their patients with a mutation in *TCF12* were of normal intelligence.

Taking into account the family history of our patient, it is unlikely that her short stature and her late‐onset hearing deficiency could be attributed to her *TCF12* haploinsufficiency. However, three patients reported with a mutation in *TCF12* (Family 31, Individual II‐1 in Supplementary Table SIII in Sharma et al. [2013] Probands 1 and 2 in Paumard‐Hernandez et al., 2014) had hearing loss suggesting that hearing loss might be an occasional feature of *TCF12*‐related syndrome, as it is of other craniosynostosis syndromes.

The facial palsy observed in our patient could have been unrelated to the microdeletion. However, one of the 38 patients reported by Sharma (Family 12, Individual I‐2 in Supplementary Table SIII) had a facial palsy [Sharma et al., 2013]. We also recently evaluated a patient (unpublished data) with a right coronal craniosynostosis, left peripheral facial palsy and a *TCF12* c.825+1G>C mutation. Five out of nine patients with a *TCF12* mutation reported by Paumard‐Hernandez et al. have facial asymmetry [Paumard‐Hernandez et al., 2014]. This suggests that facial palsy or asymmetry could be a feature of *TCF12* related conditions.

This report represents the first documented *TCF12* microdeletion and, similar to other monogenic autosomal dominant genetic conditions, demonstrates that partial or complete microdeletion of the gene accounts for a small percentage of cases. It remains to be elucidated if the microdeletion phenotype differs from the recently described point mutation associated phenotype. Additional reports would be necessary to clarify if the microdeletion phenotype differs from the point mutation associated phenotype, and confirm if there is a *TCF12* related facial gestalt. The inclusion of *TCF12* in gene panels for the investigation of intellectual disability should be considered.

## ON LINE RESOURCES

Database of Genomic Variants: http://dgv.tcag.ca/
